# Genetic polymorphisms in dopamine-related genes and smoking cessation in women: a prospective cohort study

**DOI:** 10.1186/1744-9081-3-22

**Published:** 2007-04-28

**Authors:** Thanh GN Ton, Mary Anne Rossing, Deborah J Bowen, Sengkeo Srinouanprachan, Kristine Wicklund, Federico M Farin

**Affiliations:** 1 Department of Neurology, University of Washington, Box 359775, 325 Ninth Ave, Suite 3EH70, 98195-9775 Seattle, WA, USA; 2 Department of Epidemiology, University of Washington, Box 357236, 98195-7236 Seattle, WA, USA; 3 Public Health Sciences Division, Fred Hutchinson Cancer Research Center, PO Box 19024, 98109-1024 Seattle, WA, USA; 4 Center for Ecogenetics and Environmental Health, University of Washington, 4225 Roosevelt Way NE, Suite 100, 98105-6099 Seattle, WA, USA

## Abstract

**Background:**

Genes involved in dopaminergic neurotransmission have been suggested as candidates for involvement in smoking behavior. We hypothesized that alleles associated with reduced dopaminergic neurotransmission would be more common in continuing smokers than among women who quit smoking.

**Methods:**

The study included 593 women aged 26–65 years who participated in a twelve month smoking cessation trial conducted in 1993–1994. Participants were contacted three years after the trial to obtain updated smoking history and biological specimens. Seven polymorphisms were assessed in genes involved in dopamine synthesis (tyrosine hydoxylase [*TH*]), receptor activation (dopamine receptors [*DRD2*, *DRD3*, *DRD4*]), reuptake (dopamine transporter [*SLC6A3*]), and metabolism (catechol-o-methyltransferase [*COMT*]). Smoking cessation was assessed as "short-term" quitting (abstinence for the seven days before the conclusion of the trial) and "long-term" quitting (abstinence for the six months before a subsequent interview conducted several years later).

**Results:**

We observed no association of any polymorphism with either short- or long-term quitting. Although some relative risk estimates were consistent with weak associations, either the direction of effect was opposite of that hypothesized, or results of the short- and long-term cessation endpoints differed. However, effect modification on smoking cessation was observed between DRD2 Taq1A and SLC6A3 VNTR polymorphisms, DRD3 Ser/Gly and d,1-fenfluramine, and DRD4 VNTR and d,1-fenfluramine.

**Conclusion:**

Although these results fail to support prior findings of independent associations of these polymorphisms with smoking status, our exploratory findings suggestive of gene-gene and gene-treatment interactions warrants further investigation.

## Background

Nicotine is the primary addictive component of tobacco, and its addictive effects operate through dopamine neurotransmission in the brain's mesolimbic "reward" pathway. Genes that code for constituents of the dopaminergic system are considered candidates for involvement in addictive behaviors, including smoking. Within a cohort of women who participated in a randomized smoking cessation trial, we examined the relation of polymorphisms in genes related to dopamine synthesis (tyrosine hydroxylase [*TH*]), receptor activation (*DRD2*, *DRD3*, *DRD4*), transport and synaptic reuptake (the dopamine transporter [*SLC6A3*]) and metabolism (catechol-O-methyltransferase [*COMT*]) with the ability to quit smoking.

The genes and polymorphisms were selected based on prior reports of an association with smoking behavior as well as reports of associations with other addictive behaviors such as alcoholism. We hypothesized that alleles associated with reduced dopaminergic neurotransmission (whether due to decreased dopamine production, increased metabolism, increased synaptic reuptake, or reduced receptor activation) would be less common among women who quit smoking than among continuing smokers. The *DRD2 *receptor gene, for example, is polymorphic in the promoter region where the gene can exist as an insertion or deletion variant at position -141 (rs1799732). Because the deletion variant is associated with lower promoter activity [1], we expected that the polymorphic form would presumably decrease dopamine neurotransmission and would be associated with a decreased ability to quit smoking. Similarly, downstream on the same *DRD2 *receptor gene exists a polymorphism identified by the Taq1A restriction enzyme (A1 allele) (rs1800497), which is associated with reduced receptor binding [2,3]. Because of this reduced receptor binding ability, we hypothesized that the A1 allele would also be associated with a decreased ability to quit smoking. In contrast, the single nucleotide substitution of G by A in codon 158 of the *COMT *gene resulting in an amino acid change from valine to methonine (rs4680) is associated with low enzyme activity and thermolability [4]. We hypothesized that a reduced ability of this *COMT *polymorphic enzyme to metabolize dopamine would presumably increase dopamine neurotransmission and be associated with a higher likelihood of quitting smoking.

We also selected other polymorphisms that have been associated with smoking or addictive behaviors in prior reports, and based our hypothesis of their affect on smoking cessation from existing literature. These include *DRD3 *Ser9Gly (rs6280) *DRD4 *variable number tandem repeat (VNTR) in third exon, *SLC6A3 *VNTR in 3' untranslated region, and *TH *VNTR in first intron. Table 1 summarizes the genes and polymorphisms we assessed, the possible effect of the variant allele on dopaminergic neurotransmission, and the hypothesized direction of effect on smoking cessation. Because results of prior studies of the relation between these selected polymorphisms and smoking behavior have been inconsistent, our assessment of these polymorphisms in a population of female smokers who were highly motivated to quit could contribute to a greater understanding of genetic influences on smoking cessation.

**Table 1:** Summary of candidate genes, polymorphisms, and their presumed effect on smoking cessation under the hypothesis that reduced dopaminergic neurotransmission is associated with lower likelihood to quit smoking.

Gene	Function of Gene Product	Polymorphism (Allele of interest)	Reported effects or associations of variant [reference]	Presumed effect on dopamine neuro-transmission	Expected direction of effect on smoking cessation among those with variant allele
COMT	Dopamine metabolism	Val158Met (Met)	Met associated with low enzyme activity and thermolability [4]	Increase	more likely to quit smoking
DRD2	Dopamine receptor	-141C *Ins/Del *in promoter region (Del allele)	Lower promoter activity [1]	Decrease	less likely to quit smoking
DRD2	Dopamine receptor	Taq1 A at 10 kb downstream of coding sequence (A1 allele)	Reduced receptor binding [2, 3]	Decrease	less likely to quit smoking
DRD3	Dopamine receptor	Ser9Gly in N, terminal extracellular domain (Gly allele)	High dopamine binding affinity in Gly9 homozygotes [40]	Increase	more likely to quit smoking
DRD4	Dopamine receptor	(long repeat alleles: 6, 10 repeats)	7-repeat alleles have decreased intracellular response to dopamine [41]	Decrease	less likely to quit smoking
SLC6A3	Dopamine reuptake	VNTR in 3' untranslated region (9-repeat allele)	9-repeat alleles associated with lower levels of dopamine transporter expression [42] and lower brain protein levels [43]	Increase	more likely to quit smoking
TH	Dopamine synthesis	TCAT, tetranucleotide VNTR first intron (10-repeat allele)	10-repeat allele associated with reduced HVA levels (measure of dopamine metabolism) [44]	Decrease	less likely to quit smoking

## Methods

### Study population

Eligible individuals were female residents of Washington State who had participated in a double-blinded randomized controlled trial conducted at the Fred Hutchinson Cancer Research Center (FHCRC) in Seattle, Washington in 1993 and 1994, investigating a pharmacologic agent, d,l-fenfluramine, as a component of smoking cessation treatment [5]. The 723 participants were between 26 and 65 years of age, weighed within 85–150 percent of ideal weight for height based on the Metropolitan Life Tables, and smoked more than 10 cigarettes per day at randomization. At entry into the trial, each woman completed questionnaires regarding: smoking behavior; other behaviors, including patterns of food and alcohol consumption; smoking-related beliefs; confidence in quitting; and a variety of psychological measures. All women received a self-help smoking cessation program from the American Lung Association. Women were randomized to receive either d,1-fenfluramine or placebo control in a double-blinded fashion. Quit outcome was assessed at 12-month follow-up.

For the current genetic study, we contacted clinical trial participants to request a biological specimen (blood or buccal cells) and to re-assess smoking behavior several years after completion of the trial (median, 3.3 years). Data and specimens were obtained from 593 women, 517 of whom provided blood and 76, buccal cells. DNA extraction and genotyping were conducted at the Functional Genomics Laboratory of the Center for Ecogenetics and Environmental Health at the University of Washington. The following polymorphisms were assessed: *COMT *Val158Met (rs4680), *DRD2 *-141C Ins/Del (rs1799732), *DRD2 *Taq1A (rs1800497), *DRD3 *Ser9Gly (rs6280), and VNTRs in the *DRD4*, *SLC6A3*, and *TH *genes. Laboratory personnel were blinded to smoking status and quality control samples were used to ensure accurate genotyping techniques. Primers, probes and conditions for genotyping assays are available upon request. All study activities received human subjects approval by the Fred Hutchinson Cancer Research Center Institutional Review Board.

### Outcome measurement

We assessed smoking status at two points in time. First, women were considered "short-term" quitters if they had not smoked for at least the seven day period before their 12-month follow-up in the original clinical trial. Second, we defined "long-term" quitters as those who reported smoking abstinence for at least six consecutive months immediately before their 3-year interview for the current study. We analyzed plasma samples from self-reported long-term quitters for cotinine to validate reported smoking status. Women initially categorized as long-term quitters who had a cotinine level of 14 nanograms per milliliter or greater [6] and who did not report recent use of nicotine gum and/or patch (n = 4) were re-categorized as continuing smokers.

### Statistical analysis

For VNTR polymorphisms, we categorized alleles based on number of repeats according to groupings used in prior studies. For *DRD4*, the alleles containing 2–5 repeats were considered short and alleles containing 6–10 repeats were considered long. For *SLC6A3*, alleles were categorized based on the presence or absence of a 9-repeat. For *TH*, alleles were categorized based on the presence or absence of a 10-repeat. With the exception of the *COMT *Val158Met polymorphism, for which there were a sufficient number of individuals in the homozygous variant (Met/Met) category to consider separately, we grouped individuals who were heterozygous with those who were homozygous variants for analysis. We computed relative risks (RR) of short-term and long-term smoking cessation using the Mantel-Haenszel estimator to compute crude and adjusted RRs. Ninety-five percent confidence intervals (CI) were calculated for all RR estimates. Analyses were conducted for the entire study population and also after restricting to non-Hispanic Whites. We assessed potential confounding by age, race, and intervention arm of the randomized trial, and conducted analyses separately among younger and older women. Because prior studies reported an effect modification between *SLC6A3 *and *DRD2 *Taq1A polymorphisms on smoking cessation, we examined the influence of the combination of these two polymorphisms on smoking cessation in our population. We also explored whether any of the polymorphisms of interest modified the effect of treatment intervention on smoking cessation in this population. Data management and statistical analyses were conducted using STATA Version 8 (StataCorp, College Station, TX, USA) and SAS software Version 6.12 (SAS Institute Inc., Cary, NC, USA).

## Results

Participants (n = 593) and non-participants (n = 130) did not differ on demographics, intervention arm, baseline smoking and drinking characteristics, or short-term quit status (21 percent and 19 percent, respectively, were considered quitters; *p *= 0.7). Long-term quit status was only assessed in those who participated in the long-term interview (median, 3.3 years after enrollment in clinical trial). The proportion of women randomized to d,l-fenfluramine was similar between those who did and did not quit smoking for both cessation outcomes. The majority of participants (93%) were non-Hispanic Caucasian. Compared to women who continued to smoke at either time point, those who were short- or long-term quitters had higher income and educational levels, consumed less alcohol, started smoking at a later age, smoked fewer numbers of cigarettes per day, and had smoked for a shorter period of time when assessed at enrollment in the trial (Table 2).

**Table 2:** Characteristics of 593 women by short-term and long-term smoking cessation status, Seattle, WA, 1993–1998.

	Short-term Smoking Cessation				Long-term Smoking Cessation			
		
	Quit (n = 116)		Did not quit (n = 447)		Quit (n = 93)		Did not quit (n = 500)	
Characteristic*	N	%	N	%	N	%	N	%
Race/Ethnicity								
Caucasian American	109	94.0	413	92.4	92	98.9	458	91.6
African American	3	2.6	17	3.8	0	0.0	20	4.0
Asian Pacific Islander	0	0.0	5	1.1	0	0.0	5	1.0
Hispanic & Latin American	1	0.8	0	0.0	0	0.0	2	0.2
American Indian & Native Alaskan	0	0.0	3	0.7	0	0.0	3	0.6
Other	3	2.6	9	2.0	1	1.1	12	2.4
Age (years)								
<40	42	36.2	161	36.0	35	37.6	181	36.2
40–49	47	40.5	175	39.1	40	43.0	195	39.0
50–59	24	20.7	95	21.2	17	18.2	106	21.2
≥60	3	2.6	16	3.6	1	1.1	18	3.6
Educational level (years)								
<12	4	3.5	13	2.9	2	2.2	18	3.8
= 12	34	20.1	143	33.6	25	27.8	160	33.5
>12	75	66.4	270	63.4	63	70.0	299	62.7
Refused	3		21		3		23	
Employed								
Yes	82	70.7	338	75.8	69	74.2	371	74.2
Missing	0		1					
Annual income								
≤ $25,000	22	19.1	99	22.6	14	15.1	120	24.5
$26,000–50,000	58	50.4	225	51.4	46	49.5	247	50.5
> $50,000	35	30.4	114	26.0	33	35.4	122	25.0
Refused	0		4		0		4	
Missing	1		5		0		7	
Alcohol use 1 month prior to trial								
None	40	37.0	128	33.9	35	42.2	137	33.1
1–10 Drinks	51	47.2	163	43.1	32	38.6	187	45.2
11–19 Drinks	5	4.6	32	8.5	5	6.0	33	8.0
≥ 20 Drinks	12	11.1	55	14.6	11	13.3	57	13.8
Missing	8		69		10		86	
Average cigarette use								
10–19 cigarettes/day	33	29.2	92	20.9	25	27.5	106	22.2
20–39 cigarettes/day	69	61.1	309	70.2	58	63.7	338	68.7
40–59 cigarettes/day	10	8.8	32	7.3	7	7.7	40	8.1
≥60 cigarettes/day	1	0.9	7	1.6	1	1.1	8	1.6
Missing	3		7		2		8	
Age started smoking								
≤13	6	5.2	49	11.0	4	4.3	57	11.4
14–18	66	56.9	287	64.3	52	56.5	319	63.8
19–22	32	27.6	83	18.6	30	32.6	89	17.8
≥23	12	10.3	27	6.1	6	6.5	35	7.0
Missing	0		1		1		0	
Total duration of smoking up to entry into trial								
≤9 years	4	3.4	1	1	1.1	4	0.8	
10–19 years	26	22.4	93	26	28.3	100	20.0	
20–29 years	54	46.7	192	38	41.3	222	44.4	
30–39 years	27	23.3	129	25	27.1	140	28.0	
≥40 years	5	4.3	31	2	2.2	34	6.8	
Missing	0		1	1		0		
Trial intervention arm								
Fenfluramine	56	48.3	226	51	54.8	240	48.0	

*At entry into clinical trial, unless otherwise indicated

Information on short-term quit was available for 563 women, 116 (21%) of whom were categorized as nonsmokers and 447 (79%) as smokers at this time point. For long-term cessation outcome, 93 (16%) women had quit, and 500 (84%) continued to smoke. Fifty-nine of 116 women (51%) were non-smoking at both time points. Those who had quit for the short-term were 6.9 times (95% CI: 4.7, 10.0) more likely to be non-smoking at the later time point.

Distributions of genotypes for all the polymorphisms did not deviate to any appreciable extent from expectation predicted by Hardy-Weinberg equilibrium. We observed no association between any of the individual polymorphisms examined and smoking cessation either for the short- or long-term quit outcomes (Table 3). Although, in a few instances, RR estimates appeared to be suggestive of a weak association with smoking (albeit with wide confidence intervals), either the direction of effect was in the opposite of that hypothesized or results of the short-term and long-term cessation endpoints were inconsistent. For example, while we observed a decreased likelihood of quitting in the short-term (RR = 0.9; 95 percent CI: 0.6, 1.5) and long-term (RR = 0.7; 95% CI: 0.4, 1.3) among women with two copies of the *COMT *Met allele, we had hypothesized that the lower activity Met allele would occur more commonly among women who quit smoking. While a 30% increase in likelihood of quitting in the short-term was observed among women with one or more 9-repeat alleles of the *SLC6A3 *gene (RR = 1.3; 95% CI: 0.9, 1.8), no association with long-term quitting was observed (RR = 1.1; 95% CI: 0.7, 1.6).

**Table 3:** Relation of gene polymorphisms with short-term and long-term smoking cessation among 593 women, Seattle, WA, 1993–1998.

	Short-term smoking cessation						Long-term smoking cessation					
		
	Quit		Did not quit				Quit		Did not quit			
Polymorphism	N	%	N	%	RR	95% CI	N	%	N	%	RR	95% CI
COMT Val158Met												
Val/Val	25	22	112	25	1.0	Ref	24	26	120	24	1.0	Ref
Val/Met	63	56	204	46	1.3	0.9, 2.0	49	53	234	48	1.0	0.7, 1.6
Met/Met	25	22	124	28	0.9	0.6, 1.5	19	21	136	28	0.7	0.4, 1.3
												
DRD2 Taq1A												
A2/A2	77	67	283	64	1.0	Ref	62	67	316	64	1.0	Ref
A2/A1; A1/A1	38	33	160	36	0.9	0.6, 1.3	31	33	177	36	0.9	0.6, 1.4
												
DRD2 -141C Ins/Del												
Ins/Ins	91	79	337	76	1.0	Ref	75	80	378	76	1.0	
Ins/Del; Del/Del	24	21	106	24	0.9	0.6, 1.3	18	20	117	24	0.8	0.5, 1.3
												
DRD3 Ser9Gly												
Ser/Ser	53	46	188	42	1.0	Ref	45	48	211	43	1.0	Ref
Ser/Gly; Gly/Gly	62	54	256	58	0.9	0.5, 1.2	48	52	284	57	0.8	0.6, 1.2
												
DRD4 VNTR												
Short/Short	71	63	256	59	1.0	Ref	53	58	290	60	1.0	Ref
Short/Long; Long/Long	42	37	179	41	0.9	0.6, 1.2	39	42	193	40	1.1	0.7, 1.6
												
SLC6A3 VNTR												
No 9 repeats	57	51	259	59	1.0	Ref	50	55	283	58	1.0	
One or two 9 repeats	55	49	183	41	1.3	0.9, 1.8	41	44	209	42	1.1	0.7, 1.6
												
TH VNTR												
No 10 repeats	50	43	207	46	1.0	Ref	39	42	235	47	1.0	Ref
One or two 10 repeats	66	57	240	54	1.1	0.8, 1.5	54	59	265	53	1.2	0.8, 1.7

Adjustment for race, age, or intervention arm of the randomized trial had no appreciable influence on the risk estimates. Analyses restricted to non-Hispanic Caucasian women yielded similar results, as did analyses conducted separately among younger (<50 years) and older (≤ 50 years) women. We also conducted analyses in which we compared women who were categorized as both short-term and long-term quitters to women who had not quit smoking at either of these two time points; again, we observed no relation of any of the polymorphisms assessed with the likelihood of quitting smoking. We further observed no association of any set of combinations of dopamine receptor polymorphisms with smoking cessation for either outcome (data not shown).

There was no relation between having a *SLC6A3 *9-repeat allele and short-term (RR: 0.9; 95% CI: 0.6, 1.4) or long-term (RR: 0.8; 95% CI: 0.5, 1.4) quitting among women with the more common A2/A2 genotype for *DRD2 *Taq1A. However, women with one or two copies of the *SLC6A3 *9-repeat allele who also had either the *DRD2 *Taq1A A1/A1 or A1/A2 genotypes were significantly more likely to quit in both the short- (RR = 2.5; 95% CI: 1.3, 4.7) and long-term (RR = 1.8; 95% CI: 0.9, 3.6). We also assessed the moderating effects of genotype on the pharmacologic agent, d,l-fenfluramine, offered in the clinical trial in which these women previously participated. Except for *DRD3 *Ser9Gly and *DRD4 *VNTR polymorphisms, d,1-fenfluramine did not appear to have an effect on smoking cessation in any subgroup for any of the polymorphisms we assessed (data not shown). The likelihood of quitting smoking associated with the use of d,1-fenfluramine was slightly increased for those who carried at least one Gly allele in the *DRD3 *gene (RR = 1.5; 95% CI: 0.9, 2.6), but not for those homozygous for the Ser allele (RR = 1.05; 95% CI:, 0.62, 1.78). Among those with two short-repeat alleles in the *DRD4 *VNTR, d,1-fenfluramine was significantly associated with a higher chance of quitting (RR = 1.8; 95% CI: 1.0, 3.9). Women with one or two long-repeat alleles, however, did not benefit from the intervention (RR = 0.8; 95% CI 0.4–1.4). Table 4 summarizes our findings regarding these modifying effects.

**Table 4:** Gene-gene and gene-treatment interaction for smoking cessation in 593 women, Seattle, WA, 1993–1998.

	Short-term Quit						Long-term Quit					
		
	Quit		Did not quit				Quit		Did not quit			
	N	%	N	%	RR	95% CI	N	%	N	%	RR	95% CI
	DRD2 Taq1 A2/A2											
SLC6A3												
no 9-repeats	45	60.0	164	58.2	1.0	Ref	38	62.3	181	57.6	1.0	Ref
one or two 9-repeats	39	40.0	118	41.8	0.9	0.6, 1.4	23	37.7	133	43.4	0.8	0.5, 1.4
	DRD2 Taq1 A1/A2 or A1/A1											
SLC6A3												
no 9-repeats	12	32.4	95	58.8	1.0	Ref	12	40.0	101	57.4	1.0	Ref
one or two 9-repeats	25	67.6	64	40.2	2.5	1.3, 4.7	18	60.0	75	42.6	1.8	0.9, 3.6

	DRD3 Ser/Ser											
Arm												
Placebo	26	49.1	96	51.1	1.0	Ref	23	51.1	111	52.6	1.0	Ref
D,1-fenfluramine	27	50.9	92	48.9	1.1	0.7, 1.7	22	48.9	100	47.4	1.1	0.6, 1.8
	DRD3 Ser/Gly and Gly/Gly											
Arm												
Placebo	33	53.2	124	48.4	1.0	Ref	19	39.6	146	51.4	1.0	Ref
D,1-fenfluramine	29	46.8	132	51.6	0.9	0.5, 1.3	29	60.4	138	48.6	1.5	0.9, 2.6

	DRD4 short/short											
Arm												
Placebo	32	45.1	118	46.1	1.0	Ref	18	34.0	145	50.0	1.0	Ref
D,1-fenfluramine	39	54.9	138	53.9	1.0	0.7, 1.6	35	66.0	145	50.0	1.8	1.0, 3.0
	DRD4 short/long and long/long											
Arm												
Placebo	26	61.9	97	54.2	1.0	Ref	24	61.5	106	54.9	1.0	Ref
D,1-fenfluramine	16	38.1	82	45.8	0.8	0.4, 1.4	15	38.5	87	45.1	0.8	0.4, 1.4

## Discussion

Our study has several strengths. Relative to cross-sectional studies that assess smoking status at a single time point, the cohort design of this study allowed us to focus on cessation independent of other aspects of smoking behavior (such as initiation) and to assess quitting behavior at two widely separated time points. Examining genetic influences within a group of moderate to heavy smokers who were sufficiently motivated to quit that they chose to participate in a randomized trial might be expected to enhance our ability to isolate and identify genetic effects on smoking cessation (independent of psychosocial factors related to motivation), relative to studies based on comparisons of smokers and nonsmokers. The conduct of this study within a female population, coupled with the lack of effect of the d,l-fenfluramine intervention on smoking cessation [7], minimizes the extent to which our results might be influenced by gender or treatment arm.

Nevertheless, the study is vulnerable to several potential sources of error and issues of generalizability. Some misclassification of smoking status might be expected, since determination of short-term quitting was based entirely on self-report. Long-term quitting was verified with plasma cotinine levels. However, because cotinine levels of a typical habitual smoker (200 ng/ml) [8] decline to nonsmoking values by the third day of abstinence (based on the average half-life of 16–19 hours) [9], individuals who had smoked at some point within the six month time period used to define this endpoint may still be misclassified. Furthermore, our results may not be generalizable to populations that were either excluded or not well-represented in our study such as men, women who are not within 85–150% of ideal body weight, and non-white racial or ethnic populations.

Clear and consistent findings have not emerged in research examining candidate gene polymorphisms in relation to smoking behavior. Results of studies of the relation between the *COMT *Val158Met polymorphism and smoking behavior have been inconsistent [10-12]. Several studies that have examined the relation of this polymorphism with smoking cessation or nicotine dependence among smokers participating in cessation trials reported positive findings. Collila and colleagues observed that women recruited from a smoking cessation trial who had the Met/Met genotype were more likely to have abstained from smoking for at least seven days (OR = 2.96; 95% CI: 1.07, 8.14) and for a prolonged period of time (OR = 3.23; 95% CI: 1.13, 9.20) [10]. In another report, investigators observed a positive relation between nicotine dependence and the presence of the high activity (Val) allele of *COMT *(*p *= 0.0072) in a population of male and female smokers enrolled in cessation trials; however, they were unable to replicate these findings in an independent group of trial participants [13].

Observational studies assessing the *DRD2 *Taq1A polymorphism and smoking characteristics have also had inconsistent findings. Whereas the results of some studies suggest an association between the A1 genotype and current or past smokers relative to nonsmokers, [14-17]. Studies of subjects recruited from clinical trials have also reported associations of smoking behavior with the *DRD2 *Taq1A polymorphism in non-Hispanic Caucasian populations. In a study of 134 male and female smokers from a trial of venlafaxine or placebo plus standard of care (e.g., counseling and nicotine patch), those with the A2/A2 genotype were more likely to quit smoking than smokers with at least one A1 allele [18]. In contrast, among enrollees of a clinical trial of buproprion plus counseling, women with at least one *DRD2 *Taq1 A1 allele were somewhat less likely to report smoking at 12-month follow-up compared to women who carried two A2 alleles (OR = 0.76; 95% CI: 0.56, 1.03); no such association was observed among men [19]. In a study of 600 male and female smokers enrolled in a clinical trial of befloxatone or placebo plus counseling, individuals with genotypes containing the A1 allele were less likely to sustain abstinence during the last four weeks of the treatment period (OR = 0.74; 95% CI: 0.46, 1.18) [20]. Overall, a meta-analysis of 12 studies of *DRD2 *Taq1A polymorphisms found no association between this polymorphism and smoking cessation (OR = 1.17; 95% CI: 0.89, 1.55) [21].

Opposite directions of associations with the *DRD2 *Taq1A polymorphism have been observed in some studies of ethnic populations. In a study of lung cancer, the A1/A1 genotype was associated with current smoking status among Mexican-American but not in African-American control subjects [22]. Among 332 Japanese individuals recruited from an outpatient clinic of Aichi Cancer Center, those with the A2/A2 genotype displayed a greater likelihood of having ever smoked (OR = 3.68; 95% CI: 1.50, 9.05) or were more likely to be current smokers (OR = 3.72; 95% CI: 1.23, 11.2) relative to those with the A1/A1 genotype after adjusting for sex and age [23]. Another study of Japanese individuals recruited from the same clinic partially replicated this finding. Compared to men with the A1/A1 genotype, men with the A2/A2 genotype were more likely to be current smokers (OR = 2.32; 95 percent CI: 1.02, 5.29). This association, however, was not apparent in women [24]. In a population of Chinese smokers, those with the A2/A2 genotype smoked a greater number of cigarettes per day than smokers with at least one A1 allele [25]. Among 187 healthy Koreans genotyped for the DRD2 Taq1A polymorphism, men carrying the A1 allele were more commonly smokers (*p *= 0.049) whereas women with the A1 allele were more likely to be nonsmokers (*p *= 0.018), with no association observed when men and women were pooled together [26].

Results of studies on the *SLC6A3 *VNTR, *TH *VNTR, *DRD2 *-141C Ins/Del and *DRD3 *Ser9Gly polymorphisms are either lacking or conflicting. *SLC6A3 *9-repeat genotypes were observed to correlate with non-smoking status, smoking cessation, later age of smoking initiation, and long periods of quitting in early studies [27,28], but these associations were not observed in subsequent studies [29,30]. In a meta-analysis of four studies, the *SLC6A3 *VNTR polymorphism was not associated with smoking cessation in a fixed-effects model (OR = 0.85; 95% CI: 0.68, 1.08) or random-effects model (OR = 0.89; 95% CI: 0.63, 1.28) [21]. An examination of the *TH *VNTR polymorphism found no association with the likelihood of smoking in either Caucasians or African-Americans. Among smokers, however, the TH1 allele (the longest polymorphic repeat, corresponding to the 10-repeat allele in our study) was associated with a higher smoking rate and the TH4 allele (corresponding to the 7-repeat allele) was associated with a lower smoking rate [31]. The TH4 allele was also observed to be inversely associated with nicotine dependence among adolescent smokers in two independent populations in Australia [32,33]. The *DRD2 *-141C Ins/Del polymorphism was examined in one study among a Japanese population, but was not associated with smoking status [23]. *DRD3 *Ser9Gly has not been previously examined in relation to smoking behaviors, although it has been assessed, with inconsistent results, in studies of other addictive behaviors.

Variations in the definition or timing of smoking assessment may explain, in part, some of the dissimilar results between studies. For example, Vandenberg and colleagues observed differences in the relation of smoking with the *SLC6A3 *9-repeat allele when smokers were compared to "never smokers" (i.e., individuals who had never smoked a cigarette) versus "nonsmokers" (i.e., those who had ever smoked less than 100 cigarettes) [30], suggesting that important information may be gained by distinguishing between those who have never smoked and those who have smoked less than 100 cigarettes. Furthermore, smoking cessation is a dynamic process characterized by high rates of relapse, possibly influencing the power to detect effects at different time periods. In several smoking cessation trials, the effect of time on weekly abstinence rates was appreciable: abstinence rates rose through the first several weeks and declined over time [18,20]. Even among individuals who abstain from smoking for seven months, relapse rates between 7% and 35% have been reported [34].

Gender appears to be related to smoking cessation in complex ways. Men have been reported to have lower rates of relapse relative to women [35], and smoking cessation may be more difficult for women for reasons pertaining to demographic and smoking history [36], weight control, or social support [37]. As described above, several studies have reported gender differences in the relation of the *DRD2 *Taq1 polymorphism with smoking behavior.

Other studies have observed gene-gene interactions with smoking status or cessation. Lerman and colleagues reported an interaction of the *DRD2 *Taq1A and *SLC6A3 *VNTR polymorphisms in a population of 289 smokers and 233 controls in which non-Hispanic Caucasian participants with *SLC6A3 *9-repeat genotypes were more likely to be nonsmokers relative to those without a 9-repeat allele among those with the *DRD2 *Taq1 A2/A2 genotype (*p *= 0.008). Among those with the *DRD2 *Taq1 A1 genotypes, the *SLC6A3 *9-repeat was not associated with smoking status (*p *= 0.51) [28]. These investigators also observed a gene-gene interaction between the same two polymorphisms and smoking cessation in a population of 418 smokers of European Caucasian descent enrolled in a clinical trial of bupropion [38]. In contrast to these findings, we observed an increased likelihood of smoking cessation among a different group defined by *DRD2 *Taq1A and *SLC6A3 *9-repeat genotypes: women with the *SLC6A3 *9-repeat genotypes had an increased likelihood of quitting in either the short-term or long-term, relative to women without a *SLC6A3 *9-repeat allele, only if they also possessed the *DRD2 *Taq1 A1 genotypes. There was no apparent association between the *SLC6A3 *9-repeat genotypes and smoking cessation for either time point among women with the *DRD2 *Taq1 A2/A2 genotype. The discrepancies of these various studies are not readily explainable by differences in the distribution of racial, ethnic or gender characteristics of the study populations.

In our exploration of whether d,1-fenfluramine may be more effective in certain subgroups defined by genotypes, we observed an increased likelihood of quitting smoking associated with d,1-fenfluramine among those with one or two *DRD3 *glycine alleles in the long-term (RR = 1.5; 95% CI: 0.9, 2.6), but not among smokers without the glycine allele (RR = 1.1; 95% CI: 0.6 1.8). For women with two short alleles in the *DRD4 *VNTR polymorphism, d,1-fenfluramine appeared more effective in helping them quit smoking than for women with one or two long alleles. Studies examining the influence of genotypes on d,1-fenfluramine's effectiveness in smoking cessation are not available, although a recent study has identified a more favorable outcome for bupropion treatment among European-American smokers with the *COMT *haplotypes (rs737865 and rs165599) [39]. Similar studies may be important in identifying likely responders for common smoking cessation treatments.

## Conclusion

Our results, together with the inconsistent findings of prior studies, provide little support for an independent relation of the candidate polymorphisms we assessed with smoking cessation. However, a substantial literature based on twin studies suggests that aspects of smoking behavior are highly heritable. Findings from our exploration of effect modification suggest possible important gene-gene and gene-treatment interactions for smoking cessation. Future studies examining genetic influences on smoking cessation may prove more informative if they are designed to completely characterize variation in the genes of interest, or to more effectively identify subgroups of smokers who are more genetically inclined to quit smoking or for whom certain pharmacologic interventions may be more efficacious.

## Competing interests

The author(s) declare that they have no competing interests.

## Authors' contributions

TGNT contributed to the management, analysis and interpretation of the data, drafting and revision of the manuscript. MAR was responsible for the conception and design for the study, interpretation of the data, drafting and revising the manuscript for intellectual content. DJB contributed to the conception of the study and the revision of the manuscript for intellectual content. SS was involved in the genotyping methods, and revision of the manuscript. KW was responsible for the tracking of subjects and acquisition of data as well as for the revision of manuscript. FMF supervised the genotyping methods and contributed to the revision of manuscript. All authors read and approved the final manuscript.
